# The Influence of Deep Margin Elevation and Immediate Dentin Sealing on the Fracture Strength of Premolars Restored With Indirect Inlays: An In Vitro Study

**DOI:** 10.1002/cre2.70161

**Published:** 2025-06-24

**Authors:** Lulwa E. Al‐Turki, Osamah A. Alsulimani, Khadijah M. Baik, Haytham Othmani, Naif M. Alqarni, Abdullah A. Alqarni, Raghad A. Al‐Dabbagh

**Affiliations:** ^1^ Department of Oral Maxillofacial Prosthodontics, Faculty of Dentistry King Abdulaziz University Jeddah Makkah Province Saudi Arabia; ^2^ Department of Oral Diagnostic Sciences, Faculty of Dentistry King Abdulaziz University Jeddah Makkah Province Saudi Arabia; ^3^ Choueifat Medical Center Jeddah Makkah Province Saudi Arabia; ^4^ Al‐Rahaily medical Center Jeddah Makkah Province Saudi Arabia; ^5^ Periodontics Resident King Fahad General hospital Jeddah Makkah Province Saudi Arabia

**Keywords:** deep margin elevation, dentin sealing, fracture strength, lithium disilicate inlay restorations

## Abstract

**Objective:**

To evaluate the effect of deep margin elevation (DME) and immediate dentin sealing (IDS) on the fracture strength of premolars restored with lithium disilicate inlay restorations.

**Materials and Methods:**

Standard MOD inlays with proximal box preparations extending 3 mm apical to the cementoenamel junction were prepared on forty sound premolars (*n* = 10) randomly divided into four groups: inlays without DME and without IDS (G1); inlays without DME but with IDS (G2); inlays with DME but without IDS (G3); and inlays with both techniques applied (G4). Composite resin was applied incrementally to elevate the proximal cervical margin coronally to the cementoenamel junction. For immediate dentin sealing, bonding agent was applied immediately after tooth preparation. All teeth were restored with lithium disilicate inlays and, after adhesive resin cementation, specimens were thermomechanically aged for 500 cycles at 5°–55°C and then subjected to load failure testing. Failure loads and locations were recorded and analyzed using one‐ and two‐way ANOVA with Tukey's post‐hoc testing (*α* = 0.05). Failure modes were analyzed using descriptive statistics.

**Results:**

The mean fracture loads were 565.76 ± 233.22 N, 978.47 ± 394.2 N, 974.31 ± 334.7 N, and 1108.21 ± 292.41 N for G1, G2, G3, and G4, respectively. Deep margin elevation (*p* = 0.011) and immediate dentin sealing (*p* = 0.010) were associated with significantly increased fracture loads. Fracture loads were significantly lower for G1 teeth than for G2‐G4 teeth, but there were no significant differences between G2, G3, and G4. G1 teeth showed 50% catastrophic and non‐catastrophic failures, which increased to 60% for G2 and decreased to 20% for G3 teeth. Samples with both seals and elevation (G4) had a 60% catastrophic failure rate.

**Conclusions:**

When applied individually or together, deep margin elevation and immediate dentin sealing significantly increase the fracture strength of premolars restored with indirect lithium disilicate inlays.

**Clinical Implications:**

In the challenging setting of margin elevation, studies on the effects of immediate dentin sealing have generally been limited to evaluating marginal integrity and bond strength. The findings of this In Vitro study suggest that both deep margin elevation and immediate dentin sealing protocols are likely to improve clinical outcomes of indirect lithium disilicate inlay restorations and may be considered viable options in clinical practice.

## 1 Introduction

Advances in adhesive dentistry and restorative materials now make it possible for conservative dentistry to effectively restore carious teeth (Hopp and Land 2013). However, teeth with proximal caries can be associated with subgingival proximal margins, the prosthetic restoration of which is biologically and technically challenging. When the distance between the proximal margin and crestal bone fails to preserve biological width, there may be a need to extend the crown or perform orthodontic extrusion to move the prosthetic margin away from the crestal bone and thereby avoid the negative inflammatory consequences that may injure the healthy periodontium (Lindhe et al. 2009; Robbins 2007). Even when the biologic width is preserved, the restorative dentist must still control bleeding during prosthetic procedures such as impression registration and finishing the restoration's subgingivally placed margins (Magne and Spreafico 2012). Furthermore, subgingivally placed margins preclude acquiring digital impressions to easily and quickly fabricate prosthetic restorations.

Dietschi and Spreafico (Dietschi and Spreafico 1998) introduced the technique of deep margin elevation (DME) over 20 years ago, which aims to raise the proximal deep margin with resin composite or glass ionomer restorations without surgical intervention. The DME approach, also known as coronal margin relocation (CMR), can facilitate marginal preparation and impression making and make it more predictable (Magne and Spreafico 2012; Bertoldi et al. 2018).

Delayed dentin sealing (DDS), where adhesive is applied after the provisionalization phase just before final cementation of the indirect restoration, is common when preparing indirect restorations (Qanungo et al. 2016). In immediate dentin sealing (IDS), the dentinal tubules are sealed with a dentin adhesive while still isolating the tooth, usually with a rubber dam, to prevent dentin dehydration and contamination while treating other areas in the patient's mouth (Dietschi and Spreafico 2015). IDS also provides optimal protection against sensitivity during the temporary phase, while improving bond strength and enhancing the durability and stability of the adhesive interface (Magne et al. 2005). Brigagao et al. (Brigagão et al. 2017). reported reduced resin cement bond strength to dentin when interim cement was used before dentin sealing, while Magne et al. (2005) recommended IDS to improve the bond strength of indirectly bonded restorations.

Studies on margin elevation and IDS have generally been limited to evaluating marginal integrity and bond strength (Magne and Spreafico 2012; Qanungo et al. 2016; Brigagão et al. 2017; Dietschi et al. 2002) with little data on the fracture resistance of teeth restored with indirect restorations using margin elevation with or without IDS. Therefore, this study aimed to evaluate the effect of DME with and without IDS on the fracture strength of premolars restored with lithium disilicate inlay restorations. The null hypotheses of this study were: (i) DME does not affect the fracture load of premolars restored with lithium disilicate inlay restorations; and (ii) IDS does not affect the fracture load of premolars restored with lithium disilicate inlay restorations.

## 2 Methods

**Figure 1:**
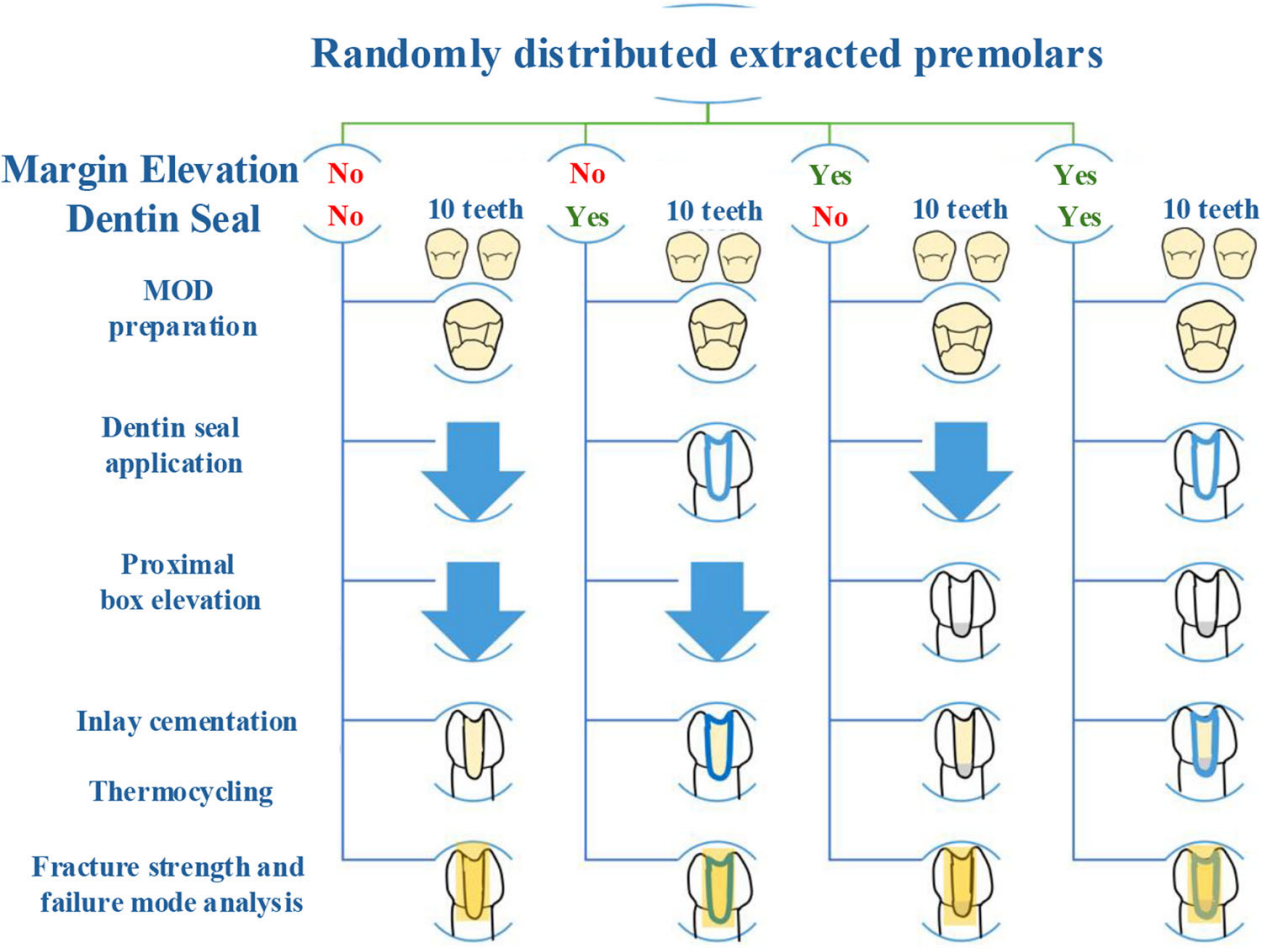
Steps involved in sample preparation and testing.

Forty extracted sound maxillary and mandibular premolars free of cracks, restorations, and endodontic treatment were mechanically cleaned with scalers and stored in 0.1% thymol solution before further processing. Selected teeth had similar buccolingual and mesiodistal crown dimensions. Teeth were randomly divided into four groups (*n* = 10 per group): inlays without DME and IDS (G1); inlays without DME but with IDS (G2); inlays with DME but without IDS (G3); inlays with DME and IDS (G4) (Figure 1).

### 2.1 Tooth Preparation

Before preparation, the cavity outline and dimensions were marked on each tooth using a standardized template. Then, teeth were securely held upright with a putty holder. For consistency and precision, new high‐speed tapered diamond burs were used for every five cavity preparations. One investigator prepared all 40 teeth. A standard step‐by‐step mesial‐occlusal‐distal (MOD) inlay cavity was prepared, including the depth, width, and angulation. The proximal box preparation was extended 3 mm apical to the cementoenamel junction and 2 mm axially. This protocol was used for all samples, and measurements were repeated regularly to ensure adherence to the predefined dimensions. After preparation, each cavity was inspected for its exact dimensions and outline. Any deviations were noted, and such samples were excluded from the study. Teeth in groups G1 and G3 were left unsealed, with dentin exposed after tooth preparation and remaining untreated until inlay cementation. Teeth in groups G2 and G4 were sealed immediately after preparation.

Each tooth was etched for 10 s with 38% phosphoric acid (Ultra‐Etch, Ultradent, South Jordan, UT, USA), rinsed with water for 10 s, air dried for 10 s, and then bonded (Universal Dentine Sealant, Ultradent) using a micro brush (Zogear, Shanghai, China) and light cured (3 M Elipar DeepCure‐S LED Curing Light, 3 M, St Paul, MN, USA) for 20 s at 10 mm. Samples in all groups were stored in saline for 24 h. The etch and bonding protocol was applied to all groups. For G3 and G4, cavities were incrementally layered with hybrid composite resin coronal to the cementoenamel junction, followed by 30 s of light curing.

### 2.2 Fabrication of Ceramic Inlays and Cementation

Elastomeric impressions were taken of all prepared teeth. Lithium disilicate inlay restorations were milled from CAD/CAM blocks (IPS e.max CAD HT A2/B 40 L, Ivoclar Vivadent, Schaan, Liechtenstein), sintered at 850°C, and glazed at 820°C. The fitting surfaces were conditioned with 10% hydrofluoric acid (Ultradent Porcelain Etch; Ultradent) for 60 s with rinsing for 60 s before being dried with oil‐free air and coating with a silane coupling agent (Ultradent Silane, Ultradent). Tooth cavities were etched with 38% phosphoric acid (Ultra‐Etch, Ultradent) for 30 s and rinsed for 15 s for all groups. Dual cure resin cement was applied (RelyX Ultimate, 3 M) to fit inlays, which were seated with a precision calibration weight of 100 g placed on top of the inlay. Excess cement was removed with a microbrush. Polymerization was carried out with LED light (3 M Elipar DeepCure‐S LED Curing Light) for 40 s on each proximal side. The teeth were then stored in water at room temperature for 2 weeks.

### 2.3 Thermocycling

Samples were aged by thermocycling for 2 min each at 5°C and 55°C for 500 cycles in deionized water baths.

### 2.4 Fracture Strength

Samples were mounted on custom‐made type 4 gypsum blocks (Moldasynt, Kulzer, Hanau, Germany) fabricated using a mold with a central hole and a 15° buccolingual tilt created by inserting a wax cylinder in the middle. After setting the gypsum, the wax was removed, and the tooth roots were coated with polyvinyl siloxane to simulate the periodontal ligament effect. Teeth were then placed in the space, which was filled with acrylic resin to resemble the alveolar bone. All samples were loaded with 2 kN compression using a Universal Testing Machine (Instron 5944, Norwood, MA, USA) at a 2 mm/min crosshead speed. Load was applied on the buccal cusp at 1 Hz. Fracture loads were recorded.

### 2.5 Mode of Failure

The type of failure was assessed using a stereomicroscope (Meiji Zoom Stereo Microscope, New York, NY, USA) to evaluate the fracture line location and extension. Failures were classified into non‐catastrophic and catastrophic fractures depending on fracture extension, where non‐catastrophic fractures featured a fracture line extending coronal to the cementoenamel junction and a catastrophic fracture featured a fracture line extending apically to the cementoenamel junction.

### 2.6 Statistical Analysis

One‐way ANOVA and Tukey's post‐hoc (*α* = 0.05) tests were used to assess for differences in fracture loads between groups (G1‐G4). Two‐way ANOVA and Tukey's post‐hoc (*α* = 0.05) tests were used to assess the effects of dentin sealing, margin elevation, and their interaction on failure loads. Failure modes were analyzed using descriptive statistics. Data were analyzed using SPSS software (IBM Corporation, Armonk, NY, USA).

## 3 Results

### 3.1 Effect of DME and IDS on Fracture Loads

**Figure 2:**
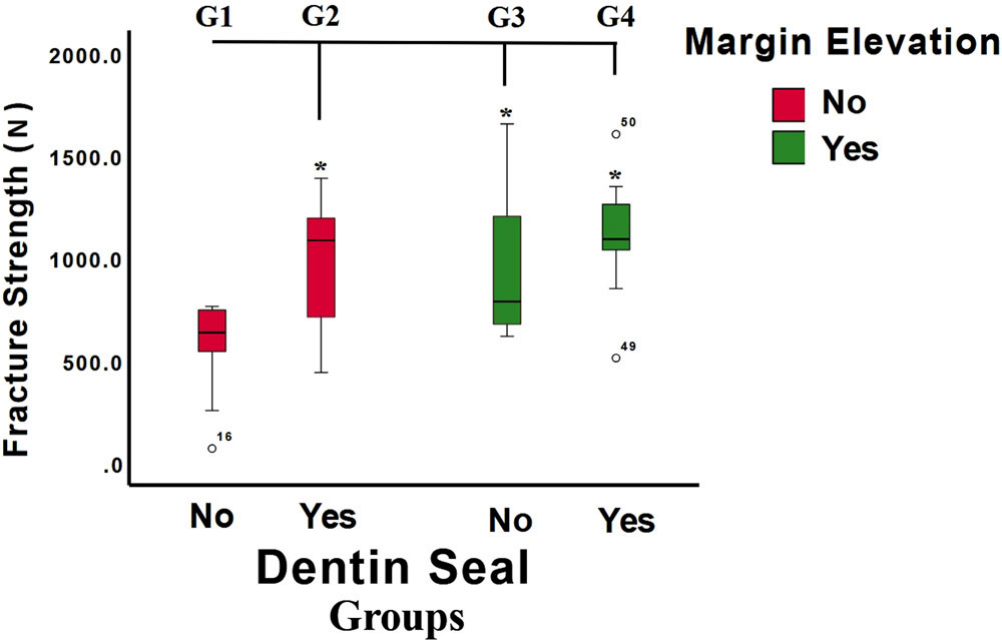
Fracture strengths of tested groups (G1‐G4). A significant difference from G1 and test groups is denoted by * at *p* < 0.05.

**Figure 3:**
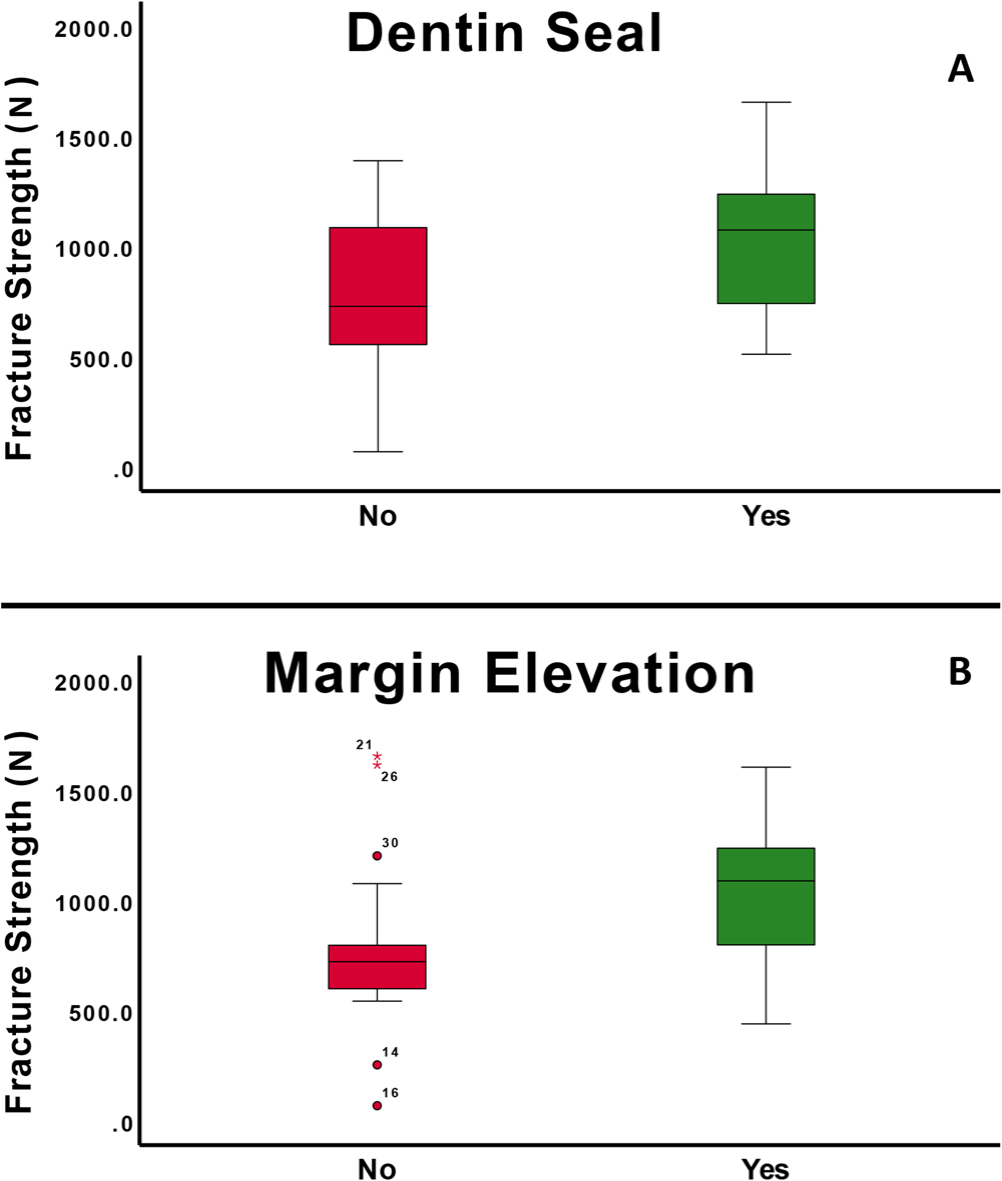
Effect of dentin sealing and margin elevation on fracture strength.

The mean fracture load of samples without DME or IDS (G1) was 565.76 ± 233.22 N. The addition of IDS (G2), DME (G3), or both (G4) significantly increased fracture loads to 978.47 ± 394.22 N, 974.31 ± 334.67 N, and 1108.21 ± 292.42 N, respectively (one‐way ANOVA; *p* = 0.003) (Table 1, Figure 2). Both DME and IDS independently increased fracture loads (two‐way ANOVA; *p* = 0.011 and *p* = 0.010, respectively, Figure 3). However, there was no significant interaction effect between DME and IDS on fracture loads (two‐wayANOVA; *p* > 0.05) (Table 1).

**Table 1:** Effect of dentin sealing and margin elevation on Fracture strength. Two‐way ANOVA and Tukey post hoc was used.

Independent variables		Fracture strength (mean ± SD)			
		Dentin seal			
		No	Yes	Total	*p* value
Elevation	No	565.76 ± 233.22 (G1)^1^	978.47 ± 394.22 (G2)^2^	772.11 ± 379.74	
	Yes	974.31 ± 334.67 (G3)^2^	1108.21 ± 292.42 (G4)^2^	1041.26 ± 313.49	
	Total	770.03 ± 350.35	1043.34 ± 344.31		0.010
	*p* value			0.011	0.176

### 3.2 Impact of DME and IDS on Failure Modes

**Figure 4:**
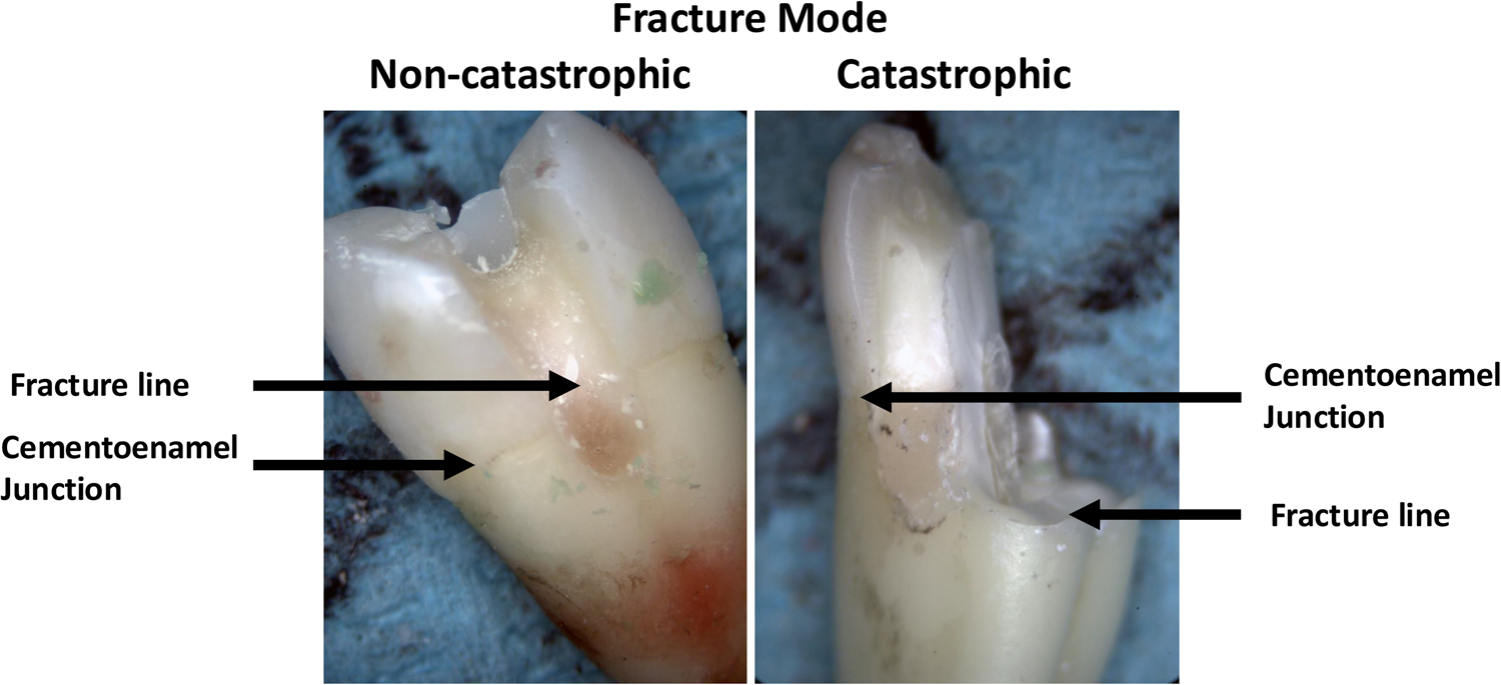
Failure modes shown by representative samples.

**Figure 5:**
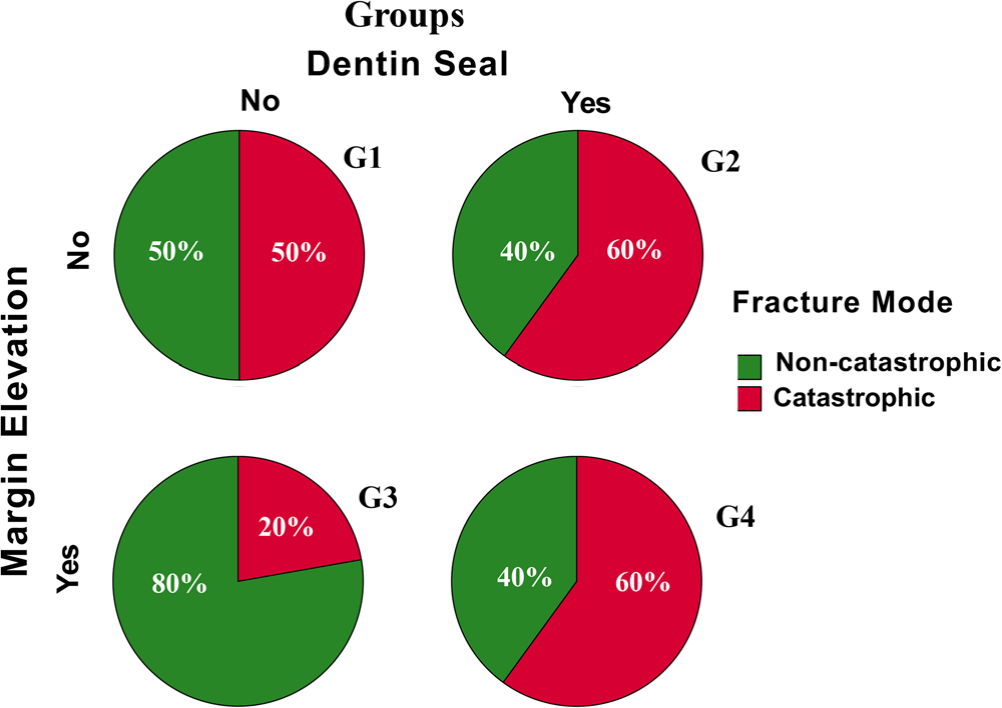
Fracture modes of the tested groups (G1‐G4).

**Figure 6:**
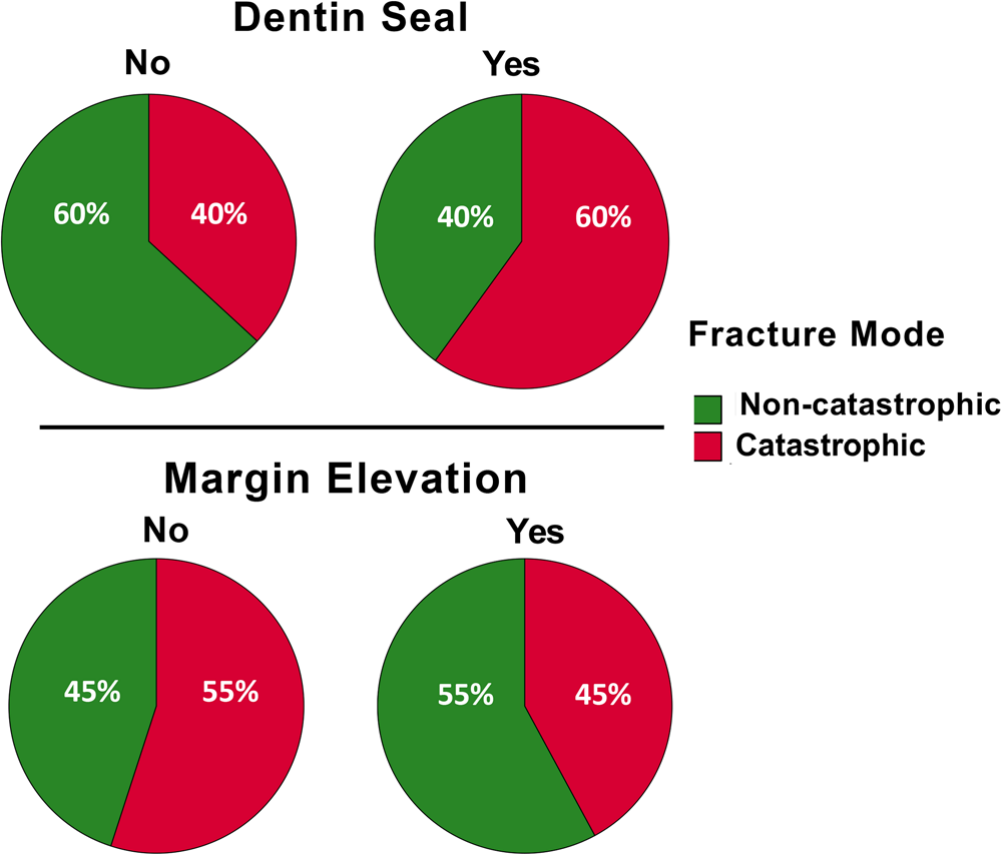
Effect of dentin sealing and margin elevation on fracture mode.

Representative examples of catastrophic and non‐catastrophic failures are shown in Figure 4. Among the groups, 50% of G1 samples failed catastrophically, compared to 60% of G2 samples and 20% of G3 samples. Samples with both DME and IDS (G4) exhibited a 60% catastrophic failure rate (Figure 5). While IDS increased the rate of catastrophic failures from 40% to 60%, DME slightly reduced catastrophic failures from 55% to 45% (Figure 6).

## 4 Discussion

This study aimed to determine whether DME with or without IDS affects the strength of premolars restored with MOD lithium disilicate inlay restorations. Our experimental results indicate that both DME and IDS significantly impact fracture strength, allowing us to reject the first and second hypotheses. However, there was no significant interaction between DME and IDS.

DME, introduced over two decades ago, has been extensively used to elevate the proximal deep margin using resin composite without surgical intervention. However, results of studies on the approach have been controversial. Bresser et al. (2020) found no effect of DME on the fracture strength of molars restored with lithium disilicate inlay and onlay restorations, and, in their finite element study, Chen et al. (2021) found only a minimal influence of DME on class II inlay restoration failure. Zhang et al. (2021) reported that margin elevation increased the fracture resistance of endodontically treated maxillary premolars restored with lithium disilicate endo‐crowns when using bulk‐filled composite compared with conventional composite. Grassi et al. (2022) found no significant effect of margin elevation on fatigue performance of molars restored with leucite‐reinforced glass ceramics and indirect resin composite inlays, with or without DME. However, their finite element analysis showed that ceramic inlays with DME reduced the maximum stress levels on both the tooth structure and ceramic inlays restorations compared with teeth restored with indirect composite inlays (Grassi et al. 2022). In their meta‐analysis, Amesti‐Amesti‐Garaizabal et al. (2019) concluded that DME does not impact the fracture strength of indirect restorations. Our results showed a significant improvement in the fracture strength of premolars restored with lithium disilicate inlays with DME.

IDS describes the immediate application of resin adhesive to freshly cut dentin after tooth preparation, followed by a second application before final cementation (Bertschinger et al. 1996; Elbishari et al. 2021). Watanabe et al. (1998) reported that temporary cementation can have adverse effects on the bond strength of resin cement to dentin, and scanning electron microscopy and x‐ray spectroscopy studies have revealed temporary cement remnants and closure of few dentinal tubules even after preconditioning dentin surfaces before application of resin (Watanabe et al. 1997). In a systematic review and meta‐analysis, Ding et al. (2023) reported that IDS effectively eliminates the adverse effects of temporary cementation, achieving a bond strength equivalent to that seen with immediate bonding. Brigagão et al. (2017) reported that IDS used before interim cement increases the microtensile bond strength of resin cement to dentin.

Magne and Kim (Magne et al. 2005) reported comparable microtensile bond strengths for indirect restorations bonded immediately after preparation by IDS and those prepared by DDS. Ishii et al. (2017) reported that IDS improved the microtensile bond strength of resin‐cemented onlay restorations, while Gresnigt et al. (2016) reported that IDS enhances lithium disilicate veneer adherence and, consequently, fracture strength. Hofsteenge et al. (2020) evaluated the fracture strengths of inlays and overlays with and without IDS and found that the fracture strength of teeth restored with overlays was significantly higher than inlays without IDS (Hofsteenge et al. 2020). However, IDS increased the fracture strength of inlays to approximate the fracture strength of overlays without IDS (Hofsteenge et al. 2020). Our results support a role for IDS in improving the fracture strength of lithium disilicate inlays.

We classified failures into non‐catastrophic fractures that extended coronal to the cementoenamel junction and catastrophic fractures that extended apical to the cementoenamel junction. There were more catastrophic fractures extending apical to the cementoenamel junction when IDS was applied to both the elevated and non‐elevated groups. This might be due to the enhanced bond strength created by IDS, which transferred the applied load apically towards the tooth structure. Alternatively, while IDS may increase strength, it may also reduce restoration toughness and predispose to sudden failure under high stress. This emphasizes the importance of balancing strength and toughness when using IDS with ceramic inlays with deep proximal margins.

By contrast, in this report, DME directed stresses coronally above the cementoenamel junction. Baldi et al. (2022) explored the normal pressure and shear stresses at the tooth restoration interface in a maxillary molar model with occluso‐proximal cavities. They used a highly filled flowable resin composite to elevate the deep proximal margins through finite element analysis created from a micro‐CT scan. They showed that DME significantly influenced the interfacial stresses, with higher stresses on the cavity floor, axial walls, and cervical margin seen when DME was not applied. Conversely, DME used with a highly filled flowable composite increased stresses only on the axial walls. Furthermore, the study reported that interfacial stresses generated at the cervical margin were influenced by the material at the cervical margins, with lower stresses seen when lithium disilicate was cemented to enamel followed by highly‐filled flowable composite liner and dentin, respectively. The study proposed a protective stress‐breaking effect of the enamel compared with the more elastic dentin and recommended the use of highly‐filled flowable composite liners in cases without cervical enamel to attenuate this effect (Baldi et al. 2022).

There is little literature on how fracture loads relate to masticatory forces. Lundgren and Laurell estimated that chewing and swallowing forces are ~100 Newtons (N), based on a maximal bite force of 320 N in habitual occlusion and an average of 37% of this force being exerted during chewing (Lundgren and Laurell 1986). In our study, the fracture strength of the tested groups exceeded any masticatory forces; however, we used static fatigue testing not only to determine whether ceramic inlays can withstand normal masticatory forces in the presence of DME and IDS but also to evaluate their relative performance under extreme conditions. Although inlays with IDS had higher fracture strength, they were still prone to catastrophic failure. This might be because IDS improved bonding of the restoration to the tooth structure, thus transferring more loading force to the tooth structure and predisposing the tooth to catastrophic failure. However, inlays with DME showed a significant increase in fracture strength and a reduction in catastrophic failure. Thus, inlays with DME might provide greater resistance to catastrophic failure under unexpectedly high loads (e.g., parafunctional habits, trauma, or accidental biting on hard objects). However, the extent of this margin varies between inlays with and without DME and IDS, and our study highlights these differences. A larger safety margin may reduce the risk of failure in challenging clinical scenarios, such as patients with bruxism or those who require restorations in high‐stress areas.

While static fracture strength provides valuable insights, long‐term clinical performance also depends on fatigue resistance and durability (Kelly et al. 2017). Inlays with DME and IDS with higher static strength may be more resistant to cyclic loading, which is critical for clinical success. In summary, while all groups in this study exceeded normal masticatory forces, the differences in static fracture strength are clinically relevant as they provide insights into the performance of ceramic inlays with DME and IDS under extreme conditions and long‐term durability. However, further studies of the fatigue behavior of ceramic inlays with DME and IDS are needed to further validate their clinical performance.

Using natural teeth with their inherent complex biologic structure may increase the variability of the results, a limitation of In Vitro studies (Bertassoni et al. 2012). Indeed, in this study, all groups showed variability in fracture strength. Our simulations may not fully replicate the complex biological environment of the oral cavity, resulting in more variability than seen in vivo. To address this, we used a standardized protocol (applied within the limitations of the study design) and controlled the experimental conditions (a standardized protocol was utilized and rigorously followed). However, to reduce this variability, we could have perhaps used other measurement techniques (e.g., cyclic loading), as static testing may result in data variability (Kelly et al. 2017). This limitation could be addressed in future work.

Our results suggest a trade‐off between strength (fracture load) and toughness (resistance to catastrophic failure). While the techniques applied increased the overall strength of the restoration [from 565.76 ± 233.22 N (without DME nor IDS) to 978.47 ± 394.2 N (with IDS without DME), 974.31 ± 334.7 N (with DME without IDS), and 1108.21 ± 292.41 N (with DME and IDS)], they might reduce its ability to absorb energy and deform before failing, leading to more catastrophic outcomes. In summary, these reported mean fracture loads support the strengthening effect of both DME and IDS, with an especially prominant effect for IDS as inferred from fracture mode analyses.

## 5 Conclusion

This study shows that both IDS and DME significantly enhance the fracture strength of premolars restored with indirect lithium disilicate inlays. Our findings suggest that DME and IDS protocols may improve clinical outcomes of indirect lithium disilicate inlay restorations. However, further studies are needed to validate these findings before clinical translation.

## Author Contributions

Conceptualization: Raghad A. Al‐Dabbagh, Lulwa E. Al‐Turki, Haytham Othmani, Naif M. Alqarni, Abdullah A. Alqarni. Methodology: Raghad A. Al‐Dabbagh, Lulwa E. Al‐Turki, Haytham Othmani, Osamah A. Alsulimani, Haytham Othmani, Naif M. Alqarni, Abdullah A. Alqarni. Formal analysis: Raghad A. Al‐Dabbagh, Lulwa E. Al‐Turki. Investigation: Raghad Al‐Dabbagh, Osamah A. Alsulimani, Khadijah M. Baik, Haytham Othmani, Naif M. Alqarni, Abdullah A. Alqarni. Resources: Raghad A. Al‐Dabbagh, Lulwa E Al‐Turki, Osamah A. Alsulimani, Khadijah M. Baik, Haytham Othmani, Naif M. Alqarni, Abdullah A. Alqarni. Writing – original draft preparation: Raghad A. Al‐Dabbagh, Lulwa E. Al‐Turki, Osamah A. Alsulimani, Khadijah M. Baik, Haytham Othmani, Naif M. Alqarni, Abdullah A. Alqarni. Writing – review, and editing: Lulwa E. Al‐Turki, Raghad A. Al‐Dabbagh, Abdullah A. Alqarni, Osamah A. Alsulimani, Khadijah M. Baik, Haytham Othmani. Visualization: Raghad A. Al‐Dabbagh, Lulwa E. Al‐Turki, Osamah A. Alsulimani, Khadijah M. Baik, Haytham Othmani, Naif M. Alqarni, Abdullah A. Alqarni. Supervision: Raghad Al‐Dabbagh. All authors have read and agreed to the published version of the manuscript.

## Conflicts of Interest

The authors declare that they have no known competing financial interests or personal relathioships that could have appeared to influence the work reported in this paper.

## Data Availability

The authors have nothing to report.
